# Regulation of HIV-1 gene expression

**DOI:** 10.1186/1742-4690-10-S1-O16

**Published:** 2013-09-19

**Authors:** Monsef Benkirane

**Affiliations:** 1 Laboratoire de Virologie Moléculaire, Institut de Génétique Humaine. CNRS UPR1142, 141 Rue de la Cardonille, 34296 Montpellier, France

## 

Gene expression is an immensely complicated process which requires fine tuning regulation involving multiple actors that have to work in a highly coordinated fashion. Indeed, each step of the transcription cycle (initiation, elongation and termination) is intimately coupled to a specif c step in pre-mRNA processing (5’ capping, splicing and 3’-end formation). Recent advances in the field highlighted the key role of the C-terminal domain (CTD) of the RNA polymerase II largest subunit in coordinating different steps of the transcription cycle and RNA processing. Transcription from the long terminal repeat (LTR) leads to RNAPII pausing and premature termination after synthesis of a short RNA, the transactivation response element (TAR). The HIV-1 transactivator protein, Tat, together with Cyclin T1 binds a bulge-loop within TAR leading to the recruitment of the super elongation complex (SEC) and allowing CDK9 to phosphorylate the RNAPII CTD and the negative transcription elongation factors NELF and the DRB-sensitivity-inducing factor DSIF/Spt4:Spt5 to overcome their negative action, licensing RNAPII for productive elongation. We will discuss how these recent advances might impact our view of how to design an efficient strategy leading to reactivation of transcriptionally silent HIV-1.

